# Brain Imaging Modalities for Cavernous Sinus Pathology With Migraine Features: A Case Report

**DOI:** 10.7759/cureus.55146

**Published:** 2024-02-28

**Authors:** Juliana Cazzaniga, Cesar Jara, Samir Ruxmohan, Jonathan Quinonez

**Affiliations:** 1 Medical School, Florida International University, Herbert Wertheim College of Medicine, Miami, USA; 2 Neurology, Larkin Community Hospital Palm Springs Campus, Hialeah, USA; 3 Neurocritical Care, University of Texas (UT) Southwestern Medical Center, Dallas, USA; 4 Osteopathic Medicine/Neurology, Larkin Community Hospital Palm Springs Campus, Hialeah, USA; 5 Addiction Medicine, Brandon Regional Hospital, Brandon, USA

**Keywords:** neurology, giant intracranial aneurysm, migraine, neuroradiology, general radiology

## Abstract

This case report delves into the unusual presentation of a 38-year-old female with a history of migraines, who initially presented with a severe right-sided headache and changes in vision in her right eye, which gradually improved. Although she had consulted with an eye specialist for these issues, she did not receive targeted treatment. This case underscores the necessity of vigilant evaluation and early intervention in a patient with seemingly benign symptoms, thereby highlighting the potential gravity of underlying conditions such as aneurysms. Our analysis and description of this case provide insights for clinicians to consider comprehensive assessment and to explore less common etiologies, resulting in improved patient outcomes.

## Introduction

The structure of a thrombosed aneurysm is characterized by clotted blood or debris accumulation. Giant aneurysms, exceeding a diameter of 2.5 cm, are relatively uncommon, constituting approximately 5-13% of all intracranial aneurysms. Notably, these giant aneurysms often manifest as masses rather than causing hemorrhages. They can result in mass effects on adjacent brain tissue, leading to cranial nerve palsies involving cranial nerves 3, 4, and 6. Spontaneous thrombosis within intracranial aneurysms, especially giant ones, is well documented [1-6]. A research study demonstrated that the ratio of aneurysmal volume to orifice contributes to intra-aneurysmal thrombus formation due to slower flow and prolonged blood retention [3,4]. Turbulent, non-laminar flow within aneurysms can foster thrombus formation, potentially resulting in downstream embolization and stroke [4]. This mechanism contributes to the heightened incidence of thrombus formation in giant intracranial aneurysms as opposed to those of standard size [1-4]. In contrast, migraines encompass various influencing factors, including genetic predisposition to specific gene variants, environmental triggers such as stimuli or hormonal fluctuations, and lifestyle-related contributors like dietary factors, psychological stress, and inadequate sleep patterns [5,6]. Additionally, recent research has suggested potential connections between certain neurological conditions and an increased risk of both migraines and intracranial aneurysms, further emphasizing the intricate nature of their interrelationship [1-4]. We present the case report of a middle-aged female who developed a migraine and was subsequently found to have an intracranial aneurysm on further examination. 

## Case presentation

**Figure 1:**
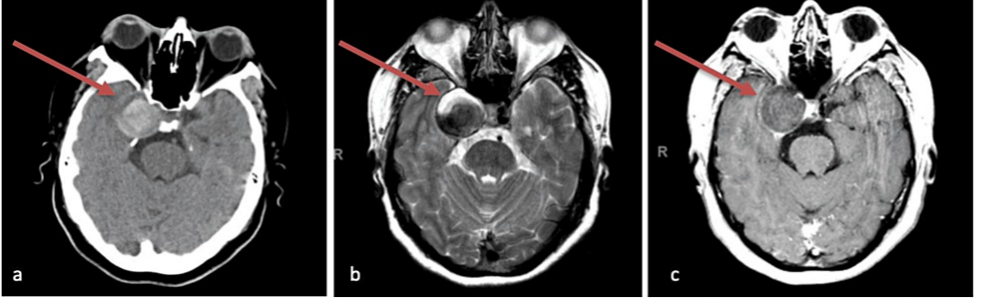
Brain CT and MRI Figure 1a shows a brain CT without contrast, demonstrating the presence of a 3.2 cm dilatation of the right internal carotid artery, indicating a thrombosed aneurysm. Figure 1b is an MRI with and without contrast. Figure 1c confirms the presence of the aneurysm.

We present the case of a 38-year-old female with a history of migraines, chronic anemia, and heavy menstrual flows who presented with a chief complaint of a constant insidious-onset severe headache for a week and alterations in vision in her right eye for three weeks. The patient stated the quality of the pain was 8/10 which has progressed to 10/10. Moreover, she stated she has loss of vision, pain with eye movements, and double vision. She later experienced nausea and vomiting. She has had migraines since age 35 and was diagnosed due to unilateral pain, photophobia, nausea, vomiting, and visual aura. Initially, the patient presented to a local community hospital but was transferred due to her requiring a higher level of care. She denied any known history of aneurysms or vascular abnormalities. According to the patient's medical records, she had a history of migraine headaches accompanied by episodes of white flashes in her right eye. The International Classification of Headache Disorders (ICHD)-3 criteria was used to diagnose migraine: visual aura for more than five minutes, nausea and vomiting occurring in succession, unilateral pain, and within 60 minutes following the aura, she felt headache [7]. These changes included an inability to track her right eye to the far right, difficulties with the peripheral visual field on the right side, particularly while driving, challenges in fully opening her right eyelid, intermittent presence of spots and floaters in her vision, as well as sporadic numbness and tingling sensations on the right side of her face, sometimes extending to instances of tingling while chewing food. Differential diagnosis includes cavernous sinus pathology. On the review of systems, the patient had vision changes, vomiting, numbness and tingling, and headache. Initial vitals include the following: blood pressure (BP), 138/88; pulse, 90; respiratory rate, 19/min; temperature (axillary), 36.9°C (98.4°F); oxygen saturation (SpO2), 95%; weight, 90.7 kg (200 lb); and height, 1.58 m (5’2”). Right-sided ptosis was observed on physical exam, accompanied by right-gaze palsy. Additionally, there was a complete restriction of eye movement in the horizontal plane, suggestive of cranial nerve 3/6 palsies. Initial lab work did not show any acute abnormality. The patient had restriction of right eye adduction while looking to the left with nystagmus that is consistent with the one-and-a-half syndrome as she has also a right-gaze palsy. The right-gaze palsy was partial. Figure 1 below shows the brain CT and MRI.

Due to the findings from the CT and MRI, neurological assessments were conducted every hour, and the head of the bed was elevated to over 30 degrees. Cardiovascular prophylaxis was initiated with a daily aspirin (81 mg) regimen. The patient was also administered 4 mg dexamethasone every six hours, and cerebral angiograms, surgical options, and non-surgical options were considered. Our team was also focused on maintaining systolic blood pressure (SBP) below 150 mmHg and administering antihypertensives as needed, including labetalol/hydralazine, to achieve and sustain the target level.

## Discussion

The presented case underscores the diagnostic complexities that can arise when seemingly innocuous symptoms, such as migraines, are accompanied by atypical manifestations [4,5]. The patient's initial complaint of a severe headache, often associated with her history of migraines, took a turn with the sudden alterations in vision in her right eye [5]. The transient loss of vision, coupled with concurrent nausea and vomiting, raised our concerns about the potential underlying pathology [2-4]. Her detailed history revealed a constellation of changes in her right eye, encompassing impaired eye movement, disturbances in peripheral vision depicted by automated visual field assessment, bilateral inability to close eyelids, and intermittent visual disturbances in the form of spots and floaters. Additionally, sensory symptoms, such as numbness and tingling extending to the right side of her face and even manifesting while chewing food, added a layer of complexity to her presentation. The patient's history of migraines, characterized by white flashes, adds an interesting dimension to the clinical picture. Migraine aura, which often includes visual disturbances, might mimic or mask other neurological conditions. This highlights the importance of not solely attributing new symptoms to pre-existing diagnoses and underscores the need for a comprehensive assessment [5]. The lack of treatment after the patient's visit to an eye doctor emphasizes the importance of a multidisciplinary approach to patient care [6]. Collaboration between various specialties, including neurology, ophthalmology, and radiology, could have potentially expedited the identification of the underlying issue [6].

From a diagnostic standpoint, the case raised suspicion for an underlying structural abnormality such as an aneurysm [6]. The patient's history of progressively worsening symptoms in her right eye, along with sensory and motor disturbances, aligns with potential neurological involvement [5,6]. Imaging modalities, such as MRI or CT angiography (CTA), are crucial in identifying vascular anomalies, including aneurysms or other potential sources of compression [6].

The management of intracranial aneurysms has been a conundrum with no precise guidelines for treatment. Management decisions regarding aneurysms, patient comorbidities, and patient preferences are made collaboratively within a multidisciplinary team. Based on results of equivalent clinical outcomes, low recurrence rates, and cost-effectiveness, surgical clipping may be preferred for anterior circulation intracranial aneurysms. Still, endovascular coiling for posterior circulation intracranial aneurysms is less invasive and linked with superior clinical outcomes [8]. Shimizu et al. conducted dose-response research and suggested that pitavastatin and rosuvastatin are the top candidates to prevent acute subarachnoid hemorrhage [9]. Similarly, aspirin holds promise as a potential therapeutic option for managing intracranial aneurysms. Aspirin, irrespective of how often or how long it is taken, demonstrated a notable and statistically significant reduction in the likelihood of both unruptured intracranial aneurysm (UIA) growth and aneurysmal subarachnoid hemorrhage in patients with UIAs [10].

Decision-making surrounding UIAs necessitates a multidisciplinary neurovascular team's consensus evaluation [3,4]. Factors including the risk of rupture, treatment-associated risks, and durability must be balanced against the patient's age, comorbidities, lifestyle, and personal preferences [4]. The overarching objective is to maximize the patient's quality-adjusted life years and minimize the risk of rupture and subarachnoid hemorrhage [5]. Decision-making tools such as the UIA treatment score consider patient-, aneurysm-, and treatment-related factors to guide recommendations [5,10-12]. Endovascular treatment, often involving coiling with or without balloon or stent assistance, remains the primary approach for most UIAs. Flow diversion stents present higher complication rates than standard coiling [5,10-12]. Surgical interventions encompass microsurgical clipping, usually standard procedures rather than complex approaches. Neurosurgical interventions entail an 8.34% complication rate and a 0.1% case fatality rate, with factors such as age, coagulopathy, and aneurysm size influencing outcomes [12]. However, no randomized controlled trial has directly compared endovascular treatment to neurosurgical intervention for UIAs [5,10-12].

## Conclusions

We underscore the importance of a comprehensive approach to diagnosis when faced with atypical symptom presentations. Our case also highlighted the significance of early intervention and multidisciplinary collaboration in cases where seemingly innocuous symptoms may hint at more severe underlying conditions. The unique interplay of migraines, evolving ocular symptoms, and neurological manifestations serves as a valuable learning point for clinicians, emphasizing the need for thorough evaluation and consideration of less common etiologies. This case report elucidates a unique presentation of cavernous sinus pathology in a 38-year-old female with a history of migraines. Initially presenting with a severe right-sided headache and changes in vision in her right eye, which gradually improved, the patient's symptoms resembled those of migraines. However, the persistence of symptoms despite consultation with an eye specialist raised suspicion of an underlying pathology. Further evaluation revealed cavernous sinus involvement, underscoring the intricate nature of symptom localization and the importance of considering less common etiologies in patients presenting with migrainous features. This case emphasizes the necessity of thorough assessment and early intervention to prevent potential complications associated with cavernous sinus pathology masquerading as migraines.
